# Case of Puerperal Fever

**Published:** 1824-09

**Authors:** H. Sanders

**Affiliations:** Surgeon, &c.


Art. VI.—*Case of Puerperal Fever.* By H. Sanders, Surgeon, &c.

Mrs. S., ætatis forty, married, was delivered of her first child, still-born, about three years, after a safe but lingering labour. During her present pregnancy, and particularly during the last three weeks, she has complained of very severe lancinating pains in the left hypochondrium; but, from a persuasion that they were connected with, and depended on, her pregnancy, medical advice was not sought.

Labour having commenced about twelve o'clock on the Sunday evening, I was sent for. On examination, I found the os uteri little better than half dilated; the membranes had ruptured, and the liquor amnii was drippling away,—indeed, had mostly escaped. The pains very frequent and severe. The funis, which was of considerable size and length, was hanging between three and four inches without the os externum, and the pulsation distinct.

The right hand was introduced into the uterus without much difficulty, and the right foot brought down into the vagina, where it was fixed by a fillet. The pains became gradually less strong, and in about an hour almost suspended, the patient having an unconquerable inclination to doze. Several injudicious attempts were made to rouse my patient, and recall the uterine efforts, by drawing the foot occasionally down by the fillet. Not finding such moderate efforts as I conceived admissible of any avail, I for the present abandoned further extractive efforts. From this time (about four in the morning) until about eleven, my patient slept: her pulse was good, and she took occasional nourishment without inconvenience. The pains now gradually advanced; but, to my great mortification, there was not a corresponding advance in the fœtus, the foot remaining in *statu quo*. The pulsation at the funis was now considerably weakened. I then, with some considerable resistance, gradually introduced my left hand, and brought down the other foot, which I likewise secured by the fillet. Delivery was then suffered to proceed in the usual way, which took place about three o'clock in the afternoon, pulsation having completely ceased in the funis; and I think it right to observe, that the child was remarkably large.

The placenta followed in less than an hour. My patient took twenty-five drops of laudanum, and fell into a comfortable sleep, which lasted some hours.

The following day (Tuesday), had passed urine freely, and without pain; after-pains trifling; lochia natural.

Wednesday.—The abdomen has become tense and swollen. The pain previously complained of has returned, with increased violence; can only lie on the right side. Neither the pulse nor tongue indicated the presence of active inflammation.—Salines and Dover's powder were prescribed, with a saline cathartic.

Thursday.—Pain somewhat increased on pressure, and the symptoms more inflammatory.—Blood was extracted from the arm to twenty-four ounces, and the salines and anodyne continued.

Friday.—Symptoms aggravated.—Twenty leeches were applied to the abdomen; blood abstracted from the arm to sixteen ounces; and the remedies continued.

Saturday.—Pain less; abdomen less tense, but still more so than natural; the blood highly cupped and buffy.—I bled again from the arm to twelve ounces; applied twelve leeches to the seat of pain; and continued the saline, without the Dover's powder.

Sunday.—The abdomen still very tense and painful; the tongue dry; respiration laborious. The blood equally inflammatory with that drawn on the preceding day.—I bled again from the arm to twelve ounces, *ad deliquium;* applied twelve leeches to the abdomen; and prescribed effervescing salines.

Monday.—Pulse 130, small, hard, and tremulous; pain increased; milk in the breasts totally disappeared, and the mammæ become quite flabby; countenance cadaverous, eyes sunken; lochia ceased altogether; the respiration hurried and anxious; pain extending down the thigh.—Twelve leeches were applied to the abdomen; a blister to the ileum; and the salines, with Dover's powder, resumed.

Tuesday.—Was sent for in great haste, supposing my patient to be *in articulo mortis.* On arriving, she had just recovered from a severe rigor: though excessively low and dejected, her countenance had assumed a more favourable aspect; she had had some refreshing sleep; her pulse about 120, and more regular; her thirst had diminished; tongue cleaner, and less dry; could bear pressure better, and had been lying on the right side for about three hours. The blister had risen well, and the pain in the thigh had nearly abated.—I continued the diaphoretic saline; applied a second blister to the sacrum; and allowed her a more generous diet.

Wednesday.—The bowels costive; had not acted since Tuesday forenoon.—Ordered a common cathartic enema.

Thursday.—Much better in general, although the pain had a little increased. The bowels had twice freely acted, from the injection.—I applied six leeches to the groin, and continued the saline.

From this time my patient became gradually convalescent, and in about six months after, on my meeting her, she told me she had again become pregnant, and feared the result; the more particularly so as she was obliged to leave London.

*Remarks.*—The attempt to recall labour-pains was injudicious, the life of the mother not being involved. Were the funis similarly situated in another instance, when endeavouring to bring down the feet, I should endeavour to hitch it on some member of the fœtus, to prevent fatal pressure on it, and to decrease the difficulty of extraction. Under similar circumstances, again, I would not be satisfied with one foot, as, when two are gained, which is done with comparatively as little pain to the mother, the fœtus is more speedily and readily expelled. In the subsequent treatment, I think much blood might have been spared, had blood to a larger amount been extracted at first, (viz. *ad deliquium.*) After the active inflammatory stage had subsided, the blisters gave evident relief. I think it but justice to add, that the circumstances of the patient were such as to preclude the possibility of a due attention to ventilation and cleanliness.

*Cleveland-court, St. James's-place: July,* 1824.

